# Bridging past and present: exploring *Cannabis* traditions in Armenia through ethnobotanical interviews and bibliographic prospecting

**DOI:** 10.1186/s42238-025-00259-x

**Published:** 2025-01-30

**Authors:** Manica Balant, Teresa Garnatje, Daniel Vitales, Marine Oganesian, Joan Vallès, Nina Stepanyan-Gandilyan, Airy Gras

**Affiliations:** 1 Institut Botànic de Barcelona (IBB), CSIC-CMCNB, Passeig del Migdia s/n, Barcelona, Catalonia 08038 Spain; 2 Laboratori de Botànica (UB), Facultat de Farmàcia i Ciències de l’Alimentació—Institut de Recerca de la Biodiversitat (IRBio), Unitat Associada al CSIC, Universitat de Barcelona, Av. Joan XXIII 27-31, Barcelona, Catalonia 08028 Spain; 3 Jardí Botànic Marimurtra-Fundació Carl Faust, Passeig Carl Faust 9, Blanes, Catalonia 17300 Spain; 4 Institute of Botany ’A. Takhtajyan’, National Academy of Sciences of the Republic of Armenia, Acharyan str. 1, Yerevan, 0040 Armenia; 5 Institut d’Estudis Catalans (IEC), Carrer del Carme 47, Barcelona, Catalonia 08001 Spain; 6 Yerevan State University, Alek Manukyan str. 1, Yerevan, 0025 Armenia

**Keywords:** Armenia, *Cannabis sativa*, Ethnobotanical survey, Traditional knowledge, Traditional uses

## Abstract

**Background:**

*Cannabis sativa* L. (Cannabaceae) has been widely used by humans throughout its history for a variety of purposes (medicinal, alimentary and other uses). Armenia, with its rich cultural history and diverse ecosystems, offers a unique context for ethnobotanical research about traditional uses of *Cannabis*. The present work aims to study and preserve the local traditional knowledge about *Cannabis* in Armenia by conducting interviews with informants and through a literature review.

**Methods:**

The first part of the dataset was gathered with ethnobotanical surveys, through questionnaires conducted with 27 informants. The second part of the data was obtained from a comprehensive bibliographic search in English, Armenian and Russian language. Since the data acquisition was different, the quantitative analyses (calculation of the number of use reports and percentages) were performed separately.

**Results:**

During the interviews 52 use reports and 3 vernacular names were recorded, while the bibliographic data from 20 references, provided us with 56 use reports and 17 *Cannabis* vernacular names, from the 5th century to 2020. Our results indicate that medicinal applications, particularly for human ailments, and fibre use have markedly dwindled, contrasting with earlier epochs. However, the *Cannabis* seeds continue to be consumed in celebrative and symbolic dishes such as *aghandz* and *tolma*.

**Conclusions:**

The recent decline in the medicinal use of *Cannabis* contrasts with earlier periods when access to pharmacological remedies was limited, and societal views of the plant were more positive. This shift can be partly attributed to the impact of legal restrictions. In contrast, the use of *Cannabis* seeds for alimentary purposed is importantly maintained nowadays. As medicinal use, fibre use has also declined, largely due to the availability of more competitive modern products. The loss of vernacular names over time, as detected in this study, also reflects the erosion of traditional knowledge, which correlates with diminishing use. Despite the small sample size and limited geographic scope, the combination of two approaches—information from contemporary informants and a systematic bibliographic review—has provided valuable insights into the changes in the traditional use of *Cannabis* in Armenia, that has not been explored in this way before.

**Supplementary Information:**

The online version contains supplementary material available at 10.1186/s42238-025-00259-x.

## Introduction

*Cannabis sativa* L. is a species belonging to the Cannabaceae family that has been widely used by humans throughout the history for a variety of purposes (Balant et al., 2021a, b), including fibre and textile uses (Andre et al. 2016), but also for numerous medicinal (Balafrej et al. 2024), food (Nanagulyan et al. 2020), and other uses (Balant et al., 2021a, b), especially in the regions of its presumed origin (Xie et al. 2023).

Several parts of this species, such as seeds, leaves, inflorescences, stems and roots, have traditionally been used for medicinal purposes since ancient times because of the effects of the interaction of plant cannabinoids, one of the types of compounds found in this species, with the human endocannabinoid system (ECS). The ECS is involved in the regulation of several physiological and cognitive processes, including the therapeutic uses for neuropsychological disorders and neurodegenerative diseases (Zou and Kumar 2018).

As a food plant, its uses in animal nutrition stand out (Muedi et al. 2024), but also in human consumption (Balant et al., 2021a, b and references therein). Regarding other (neither medicinal nor food) uses, textile ones, due to the relevant presence of long and resistant fibres in the plant’s stem, excel among a huge variety of applications (Crini et al. 2020). Among those, even very specific or relatively residual ones, such as pest control (Ona et al. 2022), are worthy of mention.

However, the most prominent and well-known uses of *Cannabis* have been those based on the psychoactive properties of its compounds, intimately linked to the medicinal field. *Cannabis* parts and products have been and are used as a recreational drug, leading to the development of numerous commercial cultivars aimed at obtaining varieties with high cannabinoid content, as well as being part of numerous rituals that are part of so-called magicoreligious uses. In this sense, although many of these uses are declining or even being lost (Balant et al., 2021a), the genus *Cannabis* continues to be the target of research from various perspectives, including the study of traditional uses, the subject of ethnobotany research (Balant et al. 2021b).

Although Armenia and Caucasus in general have been the subject of numerous ethnobotanical studies (Hovsepyan et al. 2014, 2016, 2019; Hovsepyan and Stepanyan-Gandilyan 2021; Melkumyan 1991; Nanagulyan et al. 2020; Pieroni et al. 2021; Sargsyan 2023; see also Fayvush et al. 2017; Grossheim 1952; Rivera et al. 2011; Stepanyan-Gandilyan, 2014 for syntheses), information on the traditional use of *Cannabis sativa* remains scarce (Nanagulyan et al. 2020). This is despite the plant having a long history of presence in the region, both as a cultivated and wild-growing species. Notably, Mnatsakanyan (1955) documents illustrations of this plant dating back to the 14th century. The scarcity of information on traditional uses of *Cannabis*, including basic ethnobotanical data, can largely be attributed to the stigma and legal restrictions surrounding its use. This was a common phenomenon worldwide, but especially so in the former Soviet Union, to which Armenia used to belong, where even botanists had a very restricted access and collection of wild *Cannabis* materials. So much so, that *Cannabis* samples are underrepresented in herbaria (A.A. Korobkov, pers. comm.). For these reasons, this topic can be sensitive and subject to misinformation.

In this context, the present work aims to: (i) study and preserve the local traditional knowledge about *Cannabis* in Armenia through questionnaires conducted with informants, (ii) recover the traditional uses of *Cannabis* in this area based on existing literature, and (iii) compare these two bodies of knowledge in order to evaluate the relevance of the traditional knowledge nowadays and its possible implications for the future.

## Materials and methods

### Data collection

**Fig. 1:**
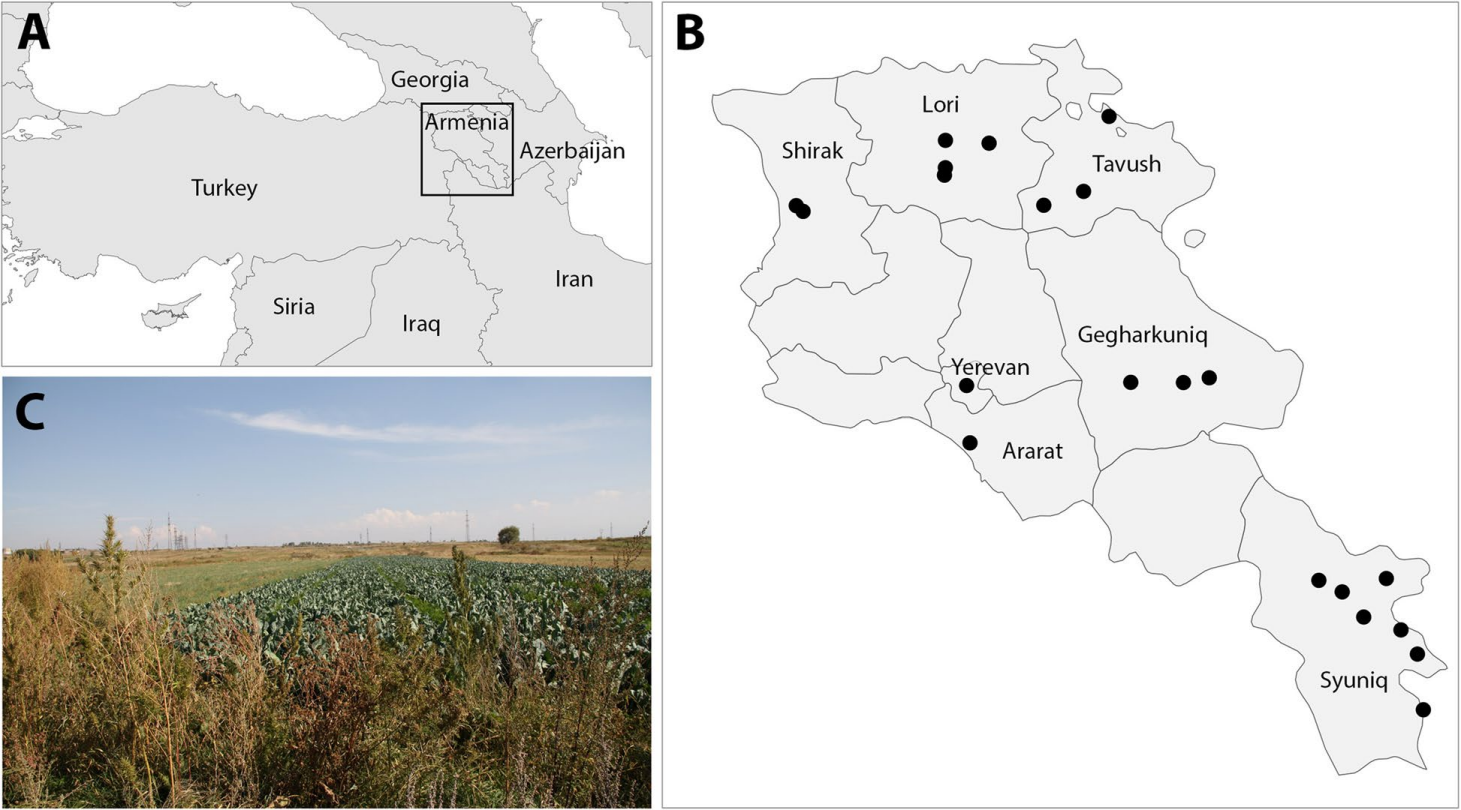
Map of the study area and adjacent countries (**A**), map of Armenia, with provinces of the ethnobotanical interviews (**B**) and a typical growing site of *Cannabis* near Gyumri (**C**)

Data were collected from two sources, implying different approaches and methods, and were therefore analysed independently. The first dataset contains information obtained from 27 individual ethnobotanical surveys, 26 of which were conducted in September 2018, in seven provinces and 21 municipalities or localities (Table 1; Fig. 1a, b, c). To the best of our knowledge, there are no formal legal requirements specifically governing the process of obtaining informed consent specific for ethnographic research in Armenia (SOCIES Expert Center, 2021). As such, oral consent was obtained in alignment with established scholarly standards and ethical principles of the International Society of Ethnobiology (2008). The data were gathered with a questionnaire centred in *Cannabis* (Supplementary material 1), which the authors developed de novo for a larger study on *Cannabis* traditional uses across the world. The initial draft of the questionnaire was developed by the research team specializing in ethnobotany and plant evolution, as part of the project framing this study. It was first written in Catalan and later translated into English for review by colleagues. The modified version has been first used in Serbia and Mongolia, with translations into local languages (i.e., Serbian and Mongolian language, respectively). These interviews served as pilot tests, but the results were not published. Following these tests, the refined questionnaire used in this study was then translated into Armenian language, with no further content modifications. The informants were recruited near wild populations of the species (though they were neither near *Cannabis* plants nor actively seeking them), with no prior screening for a minimum level of knowledge about the plant. These sites, where *Cannabis* was growing, were considered the most appropriate for conducting interviews, as it was more likely that the interviewees possessed some knowledge of the species. The locations for interviews were selected as part of a collection trip for a multidisciplinary *Cannabis* study (e.g., Balant et al. 2022). Sampling sites were chosen to reflect the genetic diversity and geographic distribution of *Cannabis* populations in Armenia as much as possible, by covering a range of ecological zones and cultivation settings. The minimum distance between sampling locations was set at 30 km in a straight line, based on the known pollen dispersal range of *Cannabis*, which generally spans 1 to 5 km (Small and Antle 2003; Nimmala et al. 2024). However, there have been reports of pollen dispersal up to 30 km (Oregon CBD, 2017), thus, the 30 km separation was deemed more appropriate. The questionnaire was not merely read, but rather integrated into the general conversation to foster a relaxed atmosphere. It is worth noting that, in many cases, potential informants declined to further engage upon hearing the topic of interview was *Cannabis*. They did not provide an explanation beyond expressing concerns about legal consequences due to the plant’s association with drugs. It is possible that those who declined might have provided additional knowledge, but the aim of this study was to present the accessible knowledge, without attempting to generalize the data to the entire country or region. The number of individuals who declined to be interviewed was not recorded, therefore only the people who agreed to participate are listed in the Supplementary material 2 of the manuscript. Additionally, one interview, following the same approach as the others, was conducted in Yerevan in January 2023. All surveys were conducted in Armenian, the shared language of the interviewers and interviewees.

**Table 1:** Number of interviews conducted in each province and gender of the informants

Province (marz)	Number of localities	Number of interviews	Number of men (%)	Number of women (%)
Ararat	1	1	1 (100)	0 (0)
Gegharquniq	3	4	2 (50)	2 (50)
Lori	4	5	2 (40)	3 (60)
Shirak	3	2 (67)	1 (33)	
Syuniq	7	9	5 (56)	4 (44)
Tavush	3	4	0 (0)	4 (100)
Yerevan	1	1	1 (100)	0 (0)
Total	21	27	13 (48)	14 (52)

During the fieldwork, herbarium vouchers were collected and deposited in the ERE herbarium of the Institute of Botany of the Armenian Academy of Sciences (vouchers’ numbers: ERE 194871–194913).

The first part of the survey includes the sociodemographic characteristics of the informants, while the second part is focused on *Cannabis* traditional uses. The personal data of the interviewees (name, surname, age, and place of residence) were recorded. However, to ensure privacy and confidentiality, all personal information not essential for the analysis was removed. During the transcription of the data into the Excel table for analysis, interviews were anonymized by assigning numerical identifiers. The number of interviews, gender of the informants and percentages are shown in Table 1, and anonymized raw data can be found in Supplementary material 2.

The second set of data was obtained from a bibliographic search using Google Scholar and Scopus databases. The search was carried out using the key words in English (i.e., *Cannabis* AND (“folk medicine” OR “traditional medicine” OR “ethnobotany” OR “traditional knowledge”) AND Armenia), Armenian (i.e.,  AND (“  ” OR “  ” OR “  ” OR “  ”) AND  ) and Russian language (i.e., конопля AND (“народное целительство” OR “традиционная медицина” OR “этноботаника” OR “традиционные знания”) AND Aрмения).

The English keyword search in Google Scholar returned 792 results, however only 3 of them contained information on ethnobotanical use of *Cannabis* in Armenia and were included in the analysis. The same key words used in Scopus database returned 7 papers, and none of them contained appropriate information and were therefore discarded. The search using keywords in Armenian returned 0 results in both Google Scholar and Scopus, while the keywords in Russian language in Google scholar returned 11 results, but with no relevant article, and 0 results in the Scopus database. Due to few relevant results, another search in Google Scholar was done using only the keyword “  ” (i.e., *Cannabis* in Armenian language), that returned 26 results, out of which 2 additional papers were included. From a total of 836 results, we were able to include information from only 5 papers. Due to the low number of papers obtained with the search in the online databases, we carried out additional searches of non-digitised bibliography. We identified key publications that contained relevant information and through the snowball method identified additional relevant literature. For this search, the Armenian-speaking territory of Nagorno-Karabakh or Artshak has also been considered (for some data on useful plants of this area, see Baloyan and Balayan 2013; and Petrov, 1940). Only the publications referring to the *Cannabis* traditional use in Armenia were included. The search returned several publications including traditional *Cannabis* use in the countries neighbouring Armenia and Caucasus region in general but could not be incorporated in the analysis since they did not explicitly mention the use was also found in Armenia. The raw data obtained with bibliographic search can be found in Supplementary material 3.

### Data analysis

Because the data acquisition was different for the two parts of the article, the quantitative analyses (calculation of the number of use reports and percentages) were performed separately in order to avoid biassed results. The first part includes the results of the interviews, so that each plant part or product use and vernacular name can be attributed to an informant (use report), while in the bibliography consulted, the number of times a use or a name have been referred to, is not mentioned. Likewise, the interviews in the first part are recent (mostly conducted in 2018) while the bibliography covers mixed old and recent references. Therefore, the two datasets are not quantitatively comparable. However, brief qualitative comparisons have been made. The limited number of interviews conducted did not allow for statistical analysis or the calculation of quantitative ethnobotanical indices for the first dataset.

## Results and discussion

### Analysis of field work data

Among the 27 informants, whose birth years range from 1929 to 1991, 13 (48.15%) were male and 14 (51.85%) were female, with an average age of 57.37 (SD 17.59) years. In total, 52 use and 28 name reports have been collected. A singular term *kanep* (pronounced with an aspirated ‘p’) in Armenian has been identified to name *Cannabis sativa*, apart from one report of the word *kanef* and one of *qol* (see below).

Most informants acknowledge that they know the plant (88.89%), most of them from their childhood, usually through its use by their parents, relatives or neighbours. All the informants, however, evoked the prohibition to grow this plant *(“police exterminate the plants*,* both of wild and cultivated hemp”*) and were even reluctant to talk about it (“*the use of hemp*,* speaking about that plant could bring problems with police”*).

The prohibition made it difficult to obtain information on traditional knowledge associated with *Cannabis*, since the first reaction of a relevant number of informants, when asked about this plant, was not to be inclined to respond. However, some informants were willing to share some more information regarding the potentially illegal uses of *Cannabis*, such as: *“after marriage*,* approximately 30 years ago*,* the relatives from Martuni brought one big sack of hemp as a present. My husband’s father ate a big quantity of not-roasted seeds and fell asleep for a long time. The households were astonished. Father-in-law liked the taste of* Cannabis *and tried to breed it in the yard*,* but the members of the family did not let him do it”* or *“I do not want to speak much about hemp*,* because using it is against the law. Note*,* that in Turkmenistan*,* where I lived some years*,* hemp is much more used as a drug”*.

**Fig. 2:**
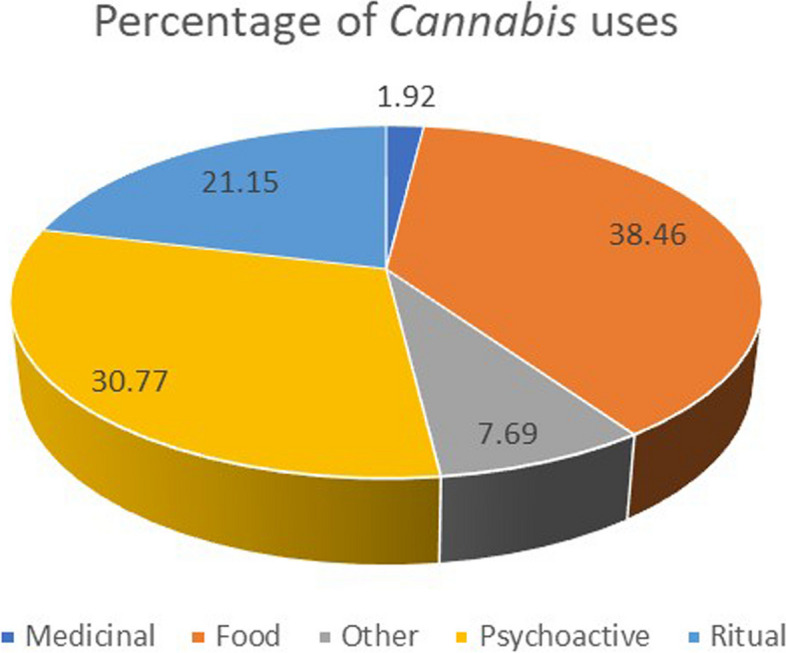
Graphical representation of the proportions of each of the medicinal, psychoactive, food, ritual and other non-food and non-medicinal uses obtained from the 27 ethnobotanical surveys carried out in Armenia

We comment below the different kinds of uses described by the informants, grouped in medicinal, psychoactive, food, ritual and other uses (non-food and non-medicinal; see Fig. 2 for more details). Despite the documented evidence in other investigations (Balant et al., 2021a), informants did not provide any accounts regarding either animal consumption as feed or use in veterinary medicine.

#### Medicinal and psychoactive uses

Out of the 52 use reports documented by the informants, only one (1.92%) pertained to medicinal use for humans. The utilised plant part consisted of seeds acquired from the market, approximately 40 years ago, which were subsequently roasted on a frying pan to enhance lactation. The mode of ingestion was direct and internal, involving the consumption of the roasted seeds. This finding aligns with existing bibliographic records (Balant et al. 2021b). Similarly, in animals, Rapetti et al. (2021) reported an increase of the milk fat content and a positive modification of the milk fatty acid composition by the ingestion of hemp seeds in dairy goats. Abbas et al. (2019) report the ingestion of seeds to increase milk production both in animals and humans. In humans, a medicinal use of powdered seeds with water was reported for boosting the immune system and enhancing sexual potency in a recent work about plants sold in the Yerevan markets (Nanagulyan et al. 2020).

Although not strictly related to medicinal premises, psychedelic *Cannabis* activities are relevant. However, discussions about them by informants may be influenced by the legal restrictions surrounding such uses (see Introduction). While 40.74% of the informants answered affirmatively to the question *“is hemp smoked in your territory?”*, when we asked them *“with what objective?”*, the answer was often *“I do not know*,* smoking hemp is against the law”* or *“I do not know*,* I was never interested on the subject”*. It is evident that these responses are incongruent with the significant volume of *Cannabis* distributed for psychoactive purposes, and such reactions are influenced by the illegality of these activities in certain countries. Only two informants specifically referred to this kind of use. One pointed out one such *Cannabis* use *“mix with tobacco and smoke”*, while another one (who explicitly asked to be maintained as anonymous), described in detail a preparation that we believe to be scarcely (if ever) reported, at least in the Caucasus. The aerial parts of the plant were used to prepare a beverage, named *malachko* (from Russian *moloko*, milk, which is a basic ingredient), to induce an altered state of consciousness. Specifically, the informant described the activity in the following terms: *“At first the feeling of knock-out*,* consciousness ‘floats’*,* sleepiness*,* laziness*,* and it is difficult to concentrate. Then that feeling goes away and the good mood comes like after smoking of hemp… For the good mood*,* sense of joy”*, indicating that *malachko*’s ingestion *“relaxes the nerves*,* calms*,* and provokes desire to communicate and dance*,* but not an aggressive attitude”*. The recipe was: *“Take a big amount of hemp unsuitable for smoking (qol) –the one that has no or smaller narcotic effect during the smoking*,* from 5–10 plants for a 20–25 l pan. Press in the pan; add milk to cover the plant material. Boil for a long time until the milk boils away up to 1/10 or 1/5 of the initial amount”*. The informant indicated that *“it has a bad smell and taste”*. Similarly, Malabadi et al. (2023) reported the use of milk to prepare *bhang*, a typical and very frequently used *Cannabis*-based drinking product in India, Pakistan, Bangladesh and nearby areas (Hourfane et al. 2023; Hussain et al. 2022; Shakil et al. 2021), indicating that using milk is a good way to extract THC.

#### Food uses

**Fig. 3:**
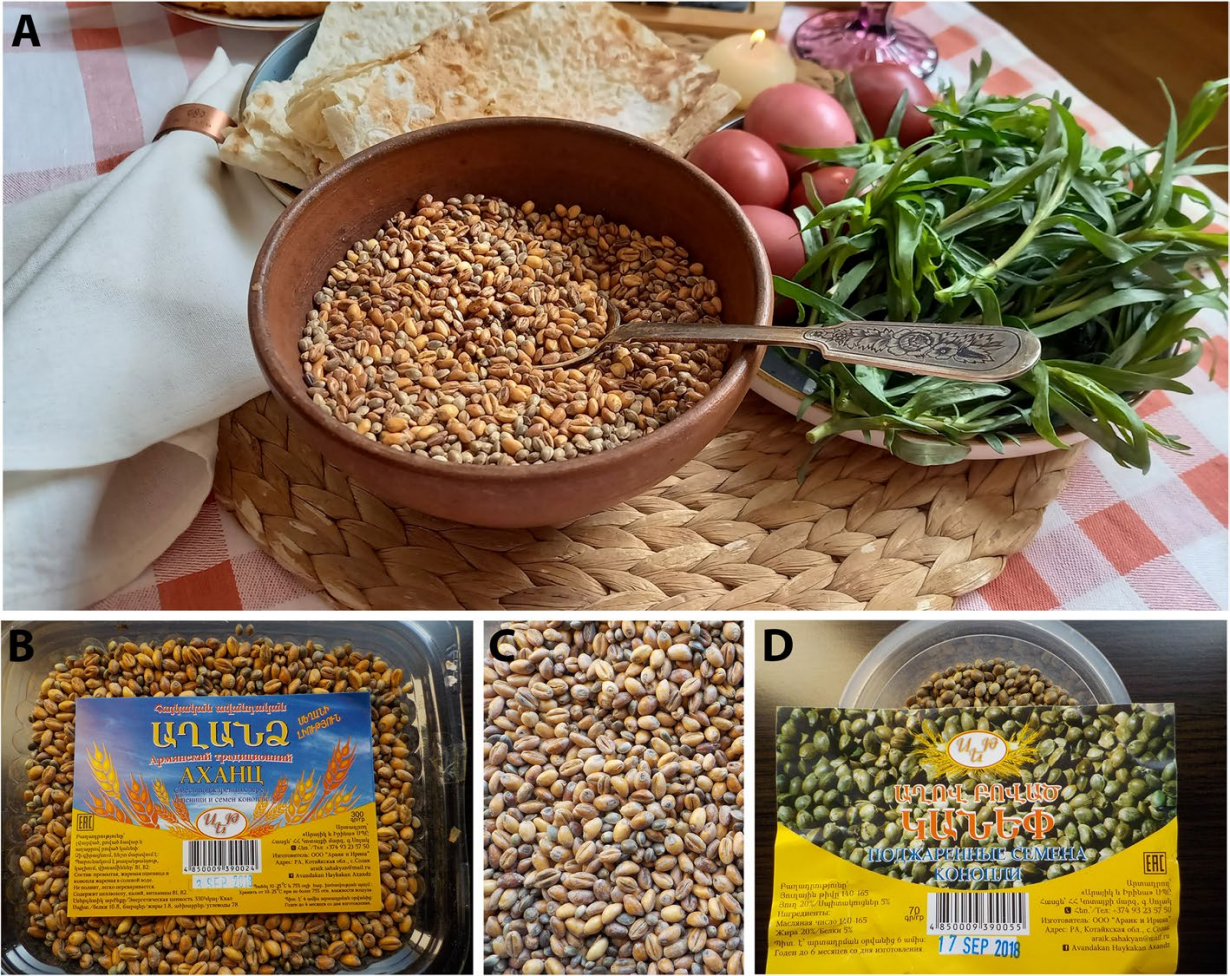
Pictures representing the typical dish *aghandz* on the Easter table (**A**), the *aghandz *(**B**,** C**), and roasted and salted *Cannabis* seeds (**D**)

Food-related applications were more frequently cited by the informants (Fig. 2), comprising 20 use reports (38.46%). In all cases, the plant part used is the seed, and in the majority of them are used to make *aghandz*, which is a kind of snack prepared with roasted wheat (*Triticum aestivum*) and *Cannabis* (Fig. 3). The preparation was described by one informant as: *“Mixing hemp seeds with seeds of wheat*,* roasting on the frying pan*,* adding salt dissolved in water*,* and continue roasting”*. *Aghandz* is not solely, but very importantly, used in the Easter period, making it also a kind of ritual food, apart from its pleasant taste and nutritious properties (see other uses in continuation). Nanagulyan et al. (2020) described the *aghandz* found in Yerevan markets as a roasted seed mix of wheat, *Cannabis* and flax (*Linum usitatissimum*).

In two instances informants reported using *Cannabis* seeds to prepare *tolma*, while in four instances, *tolma* was made with wheat (*dzavar*). An informant described it as follows: *“The seeds of* Cannabis *are put in a blender. For mincing*,* in old times*,* stones were used. Riddle through the coarse sieve to separate the husk. Dissolve in the warm water to make an oil-like mixture. Prepare* dzavarov tolma *(*tolma *with wheat instead of meat). Add the oil from the hemp to the* tolma*”*. *Tolma* is an Armenian dish consisting of spiced meats, rice (*Oryza sativa*), and herbs, typically wrapped in grapevine (*Vitis vinifera*) leaves. Traditionally, it was prepared with lamb meat, though contemporary variations feature different types of meat. With variants, it is a dish of long tradition in many Mediterranean, Balkan and western Asian territories, where it may be also named *dolma* or *sarma*, even if some nuances exist between the products elaborated under both terms (Dogan et al. 2015). The etymology of the Armenian denomination comes from the old Armenian root *toli* which means ‘grape’ (Acharian 1926), whereas the similar word *dolma*, even if used also in Greek, means ‘stuffed’ in Turkish, which can be referred both to the wrapping leaves or their content (Merriam-Webster Dictionary, 2024). Conversely to what has been recorded in Armenia, *Cannabis* seeds have not been reported as used to fill this kind of food preparation in the Balkan area (Dogan et al. 2015).

In only one case each (5.26%), the seeds are used as a flavouring *(“Roasted seeds added to the pastry (inside and outside)”*), oil *(“I have heard that*,* in old time*,* people in another village (Darbas) made oil from the seeds of hemp”*), or uncooked (*“When I was a little girl*,* they used to pick the hemp in the field*,* mash it with hands and eat on place”*). The seeds are the most used part of the plant due to the absence or a very low concentration of biologically active compounds compared to other parts of the plant (Cerino et al. 2021; Rupasinghe et al. 2020). It is also important to consider the numerous Armenian diaspora all around the World, who continues with some of the Armenian uses, customs and traditions, including those linked to plants (Hanazaki et al. 2023 and references therein), among which, for instance and related to *Cannabis*, celebrative and symbolic dishes such as *aghandz* and *tolma*, continue to be consumed.

#### Religious and other uses

**Fig. 4:**
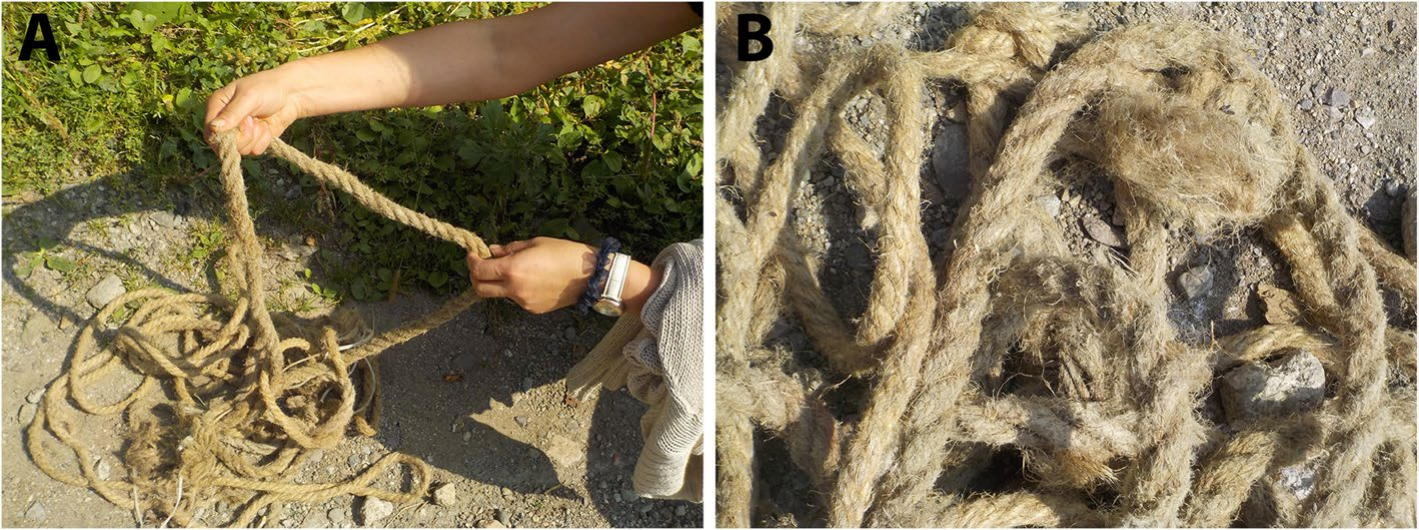
Ropes made from *Cannabis* fibres; photographs taken in Gargar (Lori marz) (**A**,** B**)

As other uses, eight (15.68%) refer to the utilisation of the stem to obtain fibres to elaborate cords, strings, ropes, scrubs and similar materials (Fig. 4). This traditional preparation is no longer frequent, but the use of these instruments has not been abandoned. Some informants mentioned that they still used a rope make from *Cannabis* fibres they have at home, even if they did not remember who and when elaborated it.

More important (48.15%) and currently still maintained, is the use of the aforementioned *aghandz* in rituals, although they are not related to the Christian religion, but, more probable, go back to the pagan times. In addition to everyday use, *aghandz* is prepared for *trndez*, a festivity of fire and sun, which is usually celebrated in February. *For atamhatik* (fest of baby’s first tooth, in Armenian ‘atam’ - tooth, ‘hatik’ - grain, seed), people add sweets to that mixture (*“on the frying pan mix with seeds of wheat and salt dissolved in water”*). For that purpose, they use colourful candies and other sweets. During the ceremony of *atamhatik*, relatives sprinkle this mixture on the head of the baby (covered with a napkin) as a sign of his/her future rich and “sweet” life. Additionally, people eat *aghandz* also on the New Year, on Easter, and on other holidays, especially on the *vardavar* - festivity of water, which is celebrated with big honour in some regions of Armenia (Fig. 3). *Tolma* is also usually prepared in winter, especially on the New Year.

Regarding the cultivation of *Cannabis*, some informants recognize that they know it is cultivated, but they also state that *“in the old times it used to be much more cultivated*,* and it was a traditional crop”*. In the present survey, the only reference reported distantly related to agricultural practices is, that *“*Cannabis *grows in the potato fields as weed*,* usually in the edge of the field”*.

#### Limitations and biases

Regarding the data obtained during our fieldwork, it is important to acknowledge certain biases. First, the sample size was limited, as legal concerns associated with certain uses of *Cannabis* led many potential informants to decline participation. Often, no specific reason was provided beyond a fact that they did not want to have legal problems. The number of individuals who declined to participate was not recorded at the time, preventing this information from being included in the analysis or considered in the study’s conclusions. Furthermore, due to these recruitment challenges, random sampling could not be implemented. The results should therefore be interpreted with some caution.

### Analysis of bibliographical data

Twenty publications and 56 entries have been recovered, which have provided 17 names and 65 uses of *Cannabis* from the 5th century (Yeznik 1994) to 2020 (Nanagulyan et al. 2020). Apart from the two oldest, the publication dates of these studies range from 1836 to 2020. Of the 56 bibliographical entries recovered, 16 contain ancient data (28.57%), 37 (66.07%), include recent information, and the remaining three (5.36%) contain mixed ancient and recent data. Most of these studies are published as books (46, 82.14%), especially the ancient references. Nine of them (16.07%) contain data from research studies, usually published in scientific journals, and the remaining one is a review (1.79%).

The data accuracy is high, meaning that each information about the plant part can be assigned to a specific use. This occurs in most cases (92.86%), but in the remaining four cases (7.143%) such information is missing.

Although all the information refers to Armenian linguistic and cultural territory, 35 entries (62.50%) correspond to the current Republic of Armenia, 20 (35.71%) to the ex-Soviet Union (when Armenia was one of its republics), and one to Nagorno-Karabakh (Artsakh in Armenian, an enclave within Azerbaijan) (1.79%).

Almost all entries refer to *Cannabis sativa* or *Cannabis* sp., only one from Nagorno-Karabakh is associated with *Cannabis ruderalis* (Petrov, 1940). However, Stoletova (1930) stated that people used both *Cannabis sativa*, *C. ruderalis* and also hybrid forms. Hovhannisyan (1945) when citing the author of the 5th century Yeznik Koghbatsi (Eznik of Kolb, arm.  ), supposed that he wrote about *Cannabis indica*. This is probably because *Cannabis* was in the past mainly considered to be a polytypic genus (Emboden 1974; Hillig 2005; Lamarck and Poiret, 1783; Schultes et al. 1974; Zhukovskiy 1971), with up to three species (*Cannabis sativa*, *C. indica* Lam. and *C. ruderalis* Janisch.), however, nowadays it most commonly accepted as a single, but very variable species *C. sativa* (Lapierre et al. 2023; McPartland 2018; Ren et al. 2021).

#### Vernacular names

Seventy-eight citations for 17 vernacular names (Table 2) in four languages (Armenian, Russian, Azerbaijani, Georgian) have been recovered from the bibliographical study of *Cannabis*. Two vernacular names attributed to *Cannabis ruderalis* have been recovered [*kinep* (Arm.  ) and *siremna* (Arm.  , in Jraberd region only)], but see the taxonomic comment above. Amasiatsi (1990), with data from 15th century, also reports vernacular names in Arabic, Persian, and Turkish languages and a big amount of information about nutritious features, medicinal use and also toxicity, as well in Armenia as in other countries, indicating different types of *Cannabis*.

**Table 2:** Vernacular names, language and transcription, number of bibliographical citations and percentage of citations for each name with respect to the total number of citations

Vernacular name	Language (transcription)	Number of bibliographical citations	%
Chetene	Azerbaijani	3	3.85%
Ganap	Armenian ( )	10	12.82%
Jetana	Azerbaijani	1	1.28%
Kanap	Armenian ( )	15	19.23%
Kanapat	Armenian ( )	1	1.28%
Kanapi	Georgian	3	3.85%
Kanaplya	Russian (Конопля)	9	11.54%
Kanaplya posevnaya	Russian (Конопля посевная)	7	8.97%
Kanep	Armenian ( )	7	8.97%
Kanepat	Armenian ( )	10	12.82%
Kaneph	Armenian	3	3.85%
Kanepuk	Armenian ( )	2	2.56%
Kenep	Armenian ( )	2	2.56%
Khin	Armenian ( )	1	1.28%
Kinep	Armenian ( )	2	2.56%
Kodzig	Armenian ( )	1	1.28%
Siremna	Armenian ( )	1	1.28%
**Total**		**78**	

#### Used plant parts and products

All entries registered are for human uses. These entries include 65 entries to used plant parts and plant products. Most of them correspond to seed (25; 38.46%), fibre (19; 29.23%) and stem (17; 26.15%).

With regard to products, it should be noted that resin is not found among those mentioned; instead, one of the most reported products is fibre, which, despite being mentioned as stem (part of the plant), is also considered a product, due to the fact that it undergoes a transformation (stems are soaked, chopped and combed to extract fibres) before being utilised for multiple applications.

Besides the three most utilised plant parts and products, others appear only once each (1.54%) in the literature corpus compiled. The ends of lateral shoots, the oil and *shira*, a white liquid containing oil (Stoletova 1930) as well as the juice from leaves and the root (Amasiatsi 1990) are examples of these minority employed parts.

#### Uses

**Fig. 5:**
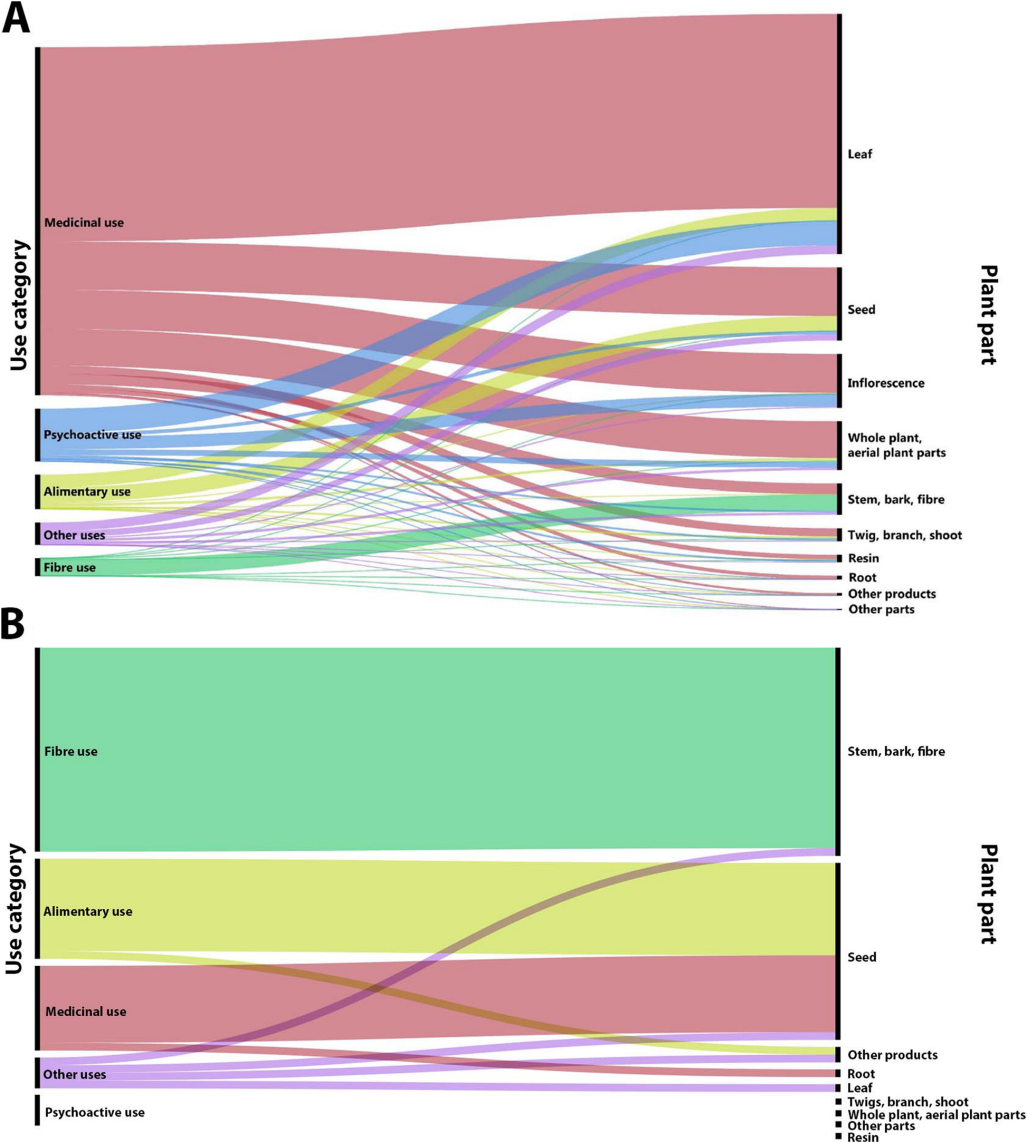
*Cannabis* uses and the plant parts used for each use category in (**A**) CANNUSE database and (**B**) uses from Armenia obtained with the current bibliographic review. The figures illustrate the relationships between use categories (left) and plant parts (right) through an alluvial chart. The flows between these two groups represent the frequencies of each plant part being used for a corresponding use category. The width of each flow is proportional to the frequency, highlighting the most commonly used plant parts for specific purposes and providing a visual summary of the connections between use categories and plant parts. The colours for individual categories in both graphs match, for easier comparison of the proportion of uses within both datasets

A total of 57 uses including medicinal (14; 24.56%), food or alimentary (16; 28.07%), fibre (21; 36.84%) and other uses (5; 8.77%), and one for psychoactive use (1.75%) (Fig. 5b). have been collected. In addition, two references about the toxicity of this species have also been recovered.

### Medicinal uses

Fourteen entries for medicinal uses were recovered from bibliography. Two of them, could not be assigned to any specific disorder, since the references concerned only refer to its medicinal use, without any specification of the disorders, or the body systems affected by these disorders. Table 3 lists the disorders and the body systems they affect, following Cook’s Economic Botany Data Collection Standard (1995). A referred use with effects to the digestive system, was as antidiarrheal and digestive, to eliminate the harmful substances from the stomach (Amasiatsi 1990) (Table 3). A possible antineoplastic effect, if we associate tumour with neoplasia, is described in this cited reference as: *“the wild hemp looks like althaea but is shorter than it. If you boil its root and make a poultice - it will help in case of hot and solid tumours and make them softer”*.

**Table 3:** Medicinal uses, system categories and specific disorders cited in the bibliography

System categories	Medicinal use recovered	Medicinal use assigned to conventional activity
Digestive system and nutritional disorders	Harmful substances in stomach	Digestive
Digestive system and nutritional disorders	Antidiarrheal	Antidiarrheal
Digestive system and nutritional disorders	Weight gain	Appetite stimulant
Endocrine system and metabolic disorders	Increase production of yellow bile	Jaundice (side effect)
Genitourinary system disorders	Increase urination	Diuretic
Immune system disorders and neoplasia	Boosting immune system	Immune system stimulant
Immune system disorders and neoplasia	Softening hard tumours	Antitumour (antineoplastic)
Nervous system and mental disorders	Enhancing sexual potency	Sexual stimulant
Nervous system and mental disorders	Speech difficulties	For aphasia
Sensory system disorders	Pain relief in ear	For otalgia (earache)
Tonic and restorative	Exhaustion recovery	Tonic
Unclassified	Medicinal use	Medicinal use

Citations about *Cannabis* toxicity and side effects are scarce and, sometimes, associated with medicinal use. Amasiatsi (1990) collected a side effect associated with medicinal use: *“eating hemp provokes an abundant urination”* (diuretic effects), *“but also the increase of yellow bile production”* (jaundice, side effect).

Most of the negative effects refer to hyposexuality (*“Hemp is a bush. Its seed is medicine*,* but also could be the cause of loss of desire”*) and the stop of the sperm emission (*“eat too much of the seeds of hemp (*kanepat, *Arm.* *) could be cause of the stop of emission of seed /sperm/“*, *“to avoid the adverse effects of seeds it is better to take them drinking cold water”*, *“if you roast and eat the seeds of* kanepat *- harm will be lesser”*) (Amasiatsi 1990). This side effect was also mentioned in Yeznik Koghbatsi, 5th century (1994): *“Its seed is medicine*,* but also could be the cause of loss of desire”*.

In some cases, problems associated with *Cannabis* (and to other plants as well) could be at the origin of the prohibitions and possible prosecutions of their cultivation, collection, commerce and utilisation, as already stated in the discussion of field work data regarding *Cannabis* psychedelic effects. Nevertheless, the use can prevail over the moral or legal difficulties, or these attitudes could not be so strict, as Hovsepyan et al. (2016) report: *“Some informants said that* Cannabis *(*Cannabis sativa *L.) is forbidden for Yezidis*,* because it has narcotic features. … However*,* these stories about lettuce*,* beans*,* hemp*,* and sunflower being forbidden for Yezidis are episodic cases; most of our informants never heard about any forbidden plants other than cabbage*,* consider these stories funny*,* and relate examples of using such apparently forbidden plants”*.

### Alimentary uses

In the bibliography consulted, there are 16 citations of alimentary uses. All the eight mixtures recorded in the literature corpus are using *Cannabis* together with other plant species, mainly for the preparation of *tolma* and *aghandz*. The most used part are the seeds and the oil extracted from them (*“seeds contain 30–40% of valuable hemp oil”*; Zhukovskiy 1982), although they are also eaten roasted *(“the seeds (*hat *or* hunt, *Arm.*  or *) of hemp*,* people roast and eat“*; Srvandztyants 1876), and boiled (*“people pounded the boiled seeds in a mortar*,* and distinguished milk /oil/*,* they use for food in the fasting days”*; Srvandztyants 1876). They are also used to prepare *aghandz* and *tolma*. Kharatyan-Arakelyan (2005) states that *“Seeds of hemp are customarily used in different regions of Armenia for preparing a delicacy called* aghandz *(Arm.* *). For preparing aghandz mix with salt roasted seeds of wheat*, Cannabis, *also raisin (*Vitis vinifera*)*,* shelled walnuts (*Arachis hypogaea*)*,* almonds (*Amygdalus communis*)*,* and*,* sometimes*,* roasted seeds of chickpea (*Cicer arietinum*). That dish is prepared for traditional holidays. From the 19th century it is served for the New year -* Amanor *(Arm.* *) holiday table*,* January 1st.”*. A variant of this dish is reported by Stoletova (1930): *“Local people roast seeds of hemp with salt*,* wheat*,* chickpea and sesame (*Sesamum indicum*). This delicacy is eaten during the festivities (as in Russia people use sunflower seeds (*Helianthus annuus*)”*. *“Some Yezidian and Kurdish people living in Armenia use seeds for preparing* tolma *(Arm.* *, as Armenians living in Martuni region of Armenia call it)”* (Hovsepyan et al. 2014).

### Fibre uses

Most of the citations for other uses refer to the fibre, used mainly as a textile and for the manufacture of ropes and twines, coarse yarn, slings, sails, oakum, etc., but also for cleaning, frost protection and soft packaging. Some examples from different sources follow: *“From bast fibre obtained true hemp -* pen’ka *(Rus. пенька) for ropes*,* slings*,* sails*,* canvas*,* oakum*,* etc.”* (Zhukovskiy 1982), and *“Fibre is used for manufacturing of coarse fabrics: for sacks*,* canvas*,* also for cords*,* ropes. The remaining short fibres are used as oakum -* paklya *(Rus. пакля)*,* for caulking of ships*,* boats*,* homes’ overlups*,* walls*,* etc.”* (Vulf 1940). Yatsenko-Khmelevskiy (1980) reported that *“Hemp fibres basically were used for ropes*,* twines*,* wrapping twines and packthread*,* part of the raw material was imported (true hemp*, pen’ka *- Rus. пенька); waste products (*oakum, paklya *- Rus. пакля) was widely used as cleaning and frost-protection material.”*. Grossheim (1952) additionally pointed out that *“The quality of the fibre obtained from the wild hemp is identical to the fibre of the cultivated hemp. It’s basically used for production of the coarse yarn and soft packaging*,* also for manufacturing of the tow/ oakum”*. In the beginning of 20th century Stoletova (1930) notes that *“hemp is rather rarely used for fibre in Armenia*,* basically for that purpose wool is in use (only in Russian settlements in Armenia it’s common to use hemp for the fibre manufacturing)”*.

### Other uses

Some minor uses are also mentioned in the recovered references, such as the magicoreligious use of the *aghandz* preparation *“for festivals Trndez and Sanct Sarqis - Surb Sarqis”* (Kharatyan-Araqelyan, 2005). Bdoyan (1972) also reports such uses: *“On the Trndez (Arm.*  , *festival of fire*, *came from pagan times) in every family people prepared and eat* aghandz *(Arm.* *)*, *which contained seeds and different dried fruits*. Aghandz *(other names -* khatsagh *(Arm.* *)* *or* khetsagh *(Arm.* *)* *had a mystical significance - seeds and fruits have role of ‘preparing’ of awakening of nature.”* and *“Different rituals with* aghandz *were connected on the one hand with idea of good harvest and fertility of the earth*,* and on other hand wish of well-being and lot of children to the young couple”*.

In agrosilvopastoral use we can find use of *Cannabis* as support for vines: *“local people cut the ends of the lateral shoots*,* the main stem remaining with hooks*,* which are used as support for grapevine”* (Stoletova 1930). For cosmetic use the leaves are *“crushed to squeeze the juice - it will clean out and strengthen the hair”* (Amasiatsi 1990). The following source does not mention the plant part, which most probably is seeds, as it is usual in Armenia: *“For the sick man who has tongue-tied /speechless/ - grease the oil of the hemp (kanapi yegh*, *Arm.*  ) *under the foot*,* it helps to loosen up”* (Srvandztyants 1876).

Another minor use, only been cited once, refers to household tasks *“*Kodzig *is a glue made from stucco*,* hemp thread and eggs*,* which was used for sticking of the broken tableware”* (Vardanyan 2004).

### Comparison of both data sets

Despite the constraints of a limited sample size and the inherent challenges pertaining to this plant in data acquisition, preliminary insights emerge at first glance. As usually occurs, the loss of plant names over time is a symptom of the erosion of traditional knowledge, correlating with diminishing utility. In the current interviews, informants have cited a single name for the plant (*kanep*) and only one informant from the Syuniq region and Yerevan mentioned another name (*kanef* and *qol*) while the literature yields a compendium of 17 vernacular designations.

Medicinal uses appear also more frequent in the literature than in the ethnobotanical survey. Historically, medicinal applications, particularly for human ailments, have markedly dwindled, contrasting with earlier epochs, when access to pharmacological remedies was constrained, and societal perceptions of the *Cannabis* were less pejorative, due to the inexistence of legal prosecution of some uses.

Conversely, some culinary practices entwined with religious observances, persist in recent ethnobotanical field work, not becoming rarer than in the literature review. This is probably due to the fact that either everyday use of *Cannabis* food or its ritual linkage are not at all related to prohibitions, since the part used is the seed, largely consumed all over the World and with no or insignificant content of psychoactive compounds.

Moreover, other uses, notably the utilisation of fibre in the fabrication of ropes, textiles, and insulation, have markedly waned due to the advent of novel materials and the obsolescence of traditional crafts.

The validity of the use of *Cannabis* for recreational purposes remains largely unassessed over time since most people do not want to talk about that in the context of an ethnobotanical interview.

Another interesting comparison that highlights the value of the work carried out in Armenia is the comparison of the results of this work with those already published on the analysis of data from the CANNUSE database (Balant et al., 2021a, b). We found 78 entries for 17 vernacular names (Table 2) while CANNUSE database lists 211 vernacular names, 40 of them in Sanskrit language (Russo 2005), being India (56 vernacular names), South Africa (34) and Pakistan (31) the countries with the most linguistic diversity.

Figure 5 shows the comparative diagrams of the uses of *Cannabis* in Armenia and those collected in the CANNUSE database. The majority of the 2330 entries of the database refer to medicinal use (75.41%), followed by psychoactive (8.35%) and alimentary use (7.29%) (Balant et al., 2021a, b). On the contrary, in Armenia the most cited category is the one for fibre uses and the medicinal is the one with the lowest percentage of the three large groups. Of the 57 uses mentioned above, only 14 are medicinal (24.56%), followed by alimentary uses (16; 28.07%), fibre uses (21; 36.84%), other uses (5; 8.77%), and only one for psychoactive use (1.75%). These differences in the importance of uses are also reflected in the most commonly used parts of the plant, which in Armenia correspond to seed (25; 38.46%), fibre (19; 29.23%) and stem (17; 26.15%), while in the CANNUSE database the most commonly used plant parts are leaf (50.51%), seed (15.38%) and inflorescence (11.35%).

## Conclusions

A comprehensive corpus of data on the traditional names and uses of *Cannabis* in Armenia was collected through ethnobotanical fieldwork and bibliographic search. One limitation of this study is the relatively small number of ethnobotanical interviews conducted, due to legal constraints and the limited geographic scope, which finally prevented more complex statistical analyses and ethnobotanical index calculations. However, we were able to combine contemporary information gathered from informants with an extensive bibliographic search, allowing us to compare changes in the traditional use of *Cannabis* over time in this region. The combination of both approaches shows a variety of medicinal (including psychoactive), alimentary or food or other different kinds of utilisations, confirming *Cannabis* as a very versatile plant from the usefulness viewpoint. This comparison between both datasets depicts a panorama in which medicinal *Cannabis*’s traditional uses have decreased, at least partly due to legal restrictions. Fibre uses are also weaker than earlier, in this case due to more competitive modern products, whereas alimentary consumption, basically concerning seeds, is importantly maintained nowadays. To sum up, local knowledge on *Cannabis* in Armenia is not negligible and may be eroded but is far from being lost.

## Supplementary Information

Supplementary Material 1.

Supplementary Material 2.

Supplementary Material 3.

## Data Availability

All data generated or analysed during this study are included in this published article and its supplementary information files.

## References

[CR1] Abbas Q, Hussain AAA, Khan SW, Hussain AAA, Shinwari S, Hussain AAA, et al. Floristic Diversity, ethnobotany and Traditional Recipes of Medicinal Plants of Maruk Nallah, Haramosh Valley, District Gilgit, Gilgit Baltistan. Proc Pakistan Acad Sci B Life Environ Sci. 2019;56:97–112.

[CR2] Acharian H. Armenian etymological dictionary. Volume 6. Publishing House of the Yerevan State University; 1926. (In Armenian).

[CR3] Amasiatsi A. Useless for Ignoramuses. Translated. Moscow: Nauka Publ., Scientific legacy, 13, Moscow; 1990. (In Russian).

[CR4] Andre CM, Hausman J-F, Guerriero G. *Cannabis sathea*plant Plant of the Thousanonenmoleculesecules. Front Plant Sci. 2016;7:19. 10.3389/fpls.2016.00019. 26870049 10.3389/fpls.2016.00019 PMC4740396

[CR5] Balafrej T, Skalli S, Benaich S, Bessi A, Mekkaoui A, El, Boukour B, et al. An ethnobotanical survey on the therapeutic use of *Cannabis sativa* L. in the province of Taounate, Morocco. Ethnobot Res Appl. 2024;28:1–14. 10.32859/era.28.26.1-14.

[CR6] Balant M, Gras A, Francisco G, Garnatje T, Vallès J, Vitales D. CANNUSE, a database of traditional *Cannabis* uses—an opportunity for new research. Database. 2021a;2021:baab024. 10.1093/database/baab024. 33942873 10.1093/database/baab024 PMC8087868

[CR7] Balant M, Gras A, Ruz M, Vallès J, Vitales D, Garnatje T. Traditional uses of *Cannabis*: an analysis of the CANNUSE database. J Ethnopharmacol. 2021b;114362. 10.1016/j.jep.2021.114362. 10.1016/j.jep.2021.114362 34171396

[CR8] Balant M, González Rodríguez R, Garcia S, Garnatje T, Pellicer J, Vallès J, et al. Novel insights into the nature of Intraspecific Genome Size Diversity in *Cannabis sativa* L. Plants. 2022;11:2736. 10.3390/plants11202736. 36297761 10.3390/plants11202736 PMC9607409

[CR9] Baloyan SA, Balayan KV. Medicinal plants of Nagorno-Karabakh. Takhtajania. 2013;2:122–30. (In Russian).

[CR10] Bdoyan VH. Land-farming culture in Armenia. Publishing House of the Academy of Sciences of the arm. SSR, Yerevan; 1972. (In Armenian).

[CR11] Cerino P, Buonerba C, Cannazza G, D’Auria J, Ottoni E, Fulgione A, et al. A review of Hemp as Food and Nutritional supplement. Cannabis Cannabinoid Res. 2021;6:19–27. 10.1089/can.2020.0001. 33614949 10.1089/can.2020.0001 PMC7891210

[CR12] Cook FEM. Economic botany data collection standard. Royal Botanic Gardens Kew. 1995. 10.1111/j.1756-1051.1997.tb00317.x.

[CR13] Crini G, Lichtfouse E, Chanet G, Morin-Crini N. Applications of hemp in textiles, paper industry, insulation and building materials, horticulture, animal nutrition, food and beverages, nutraceuticals, cosmetics and hygiene, medicine, agrochemistry, energy production and environment: a review. Environ Chem Lett. 2020;18:1451–76. 10.1007/s10311-020-01029-2.

[CR14] Dogan Y, Nedelcheva A, Łuczaj Ł, Drăgulescu C, Stefkov G, Maglajlić A, et al. Of the importance of a leaf: the ethnobotany of sarma in Turkey and the Balkans. J Ethnobiol Ethnomed. 2015;11:26. 10.1186/s13002-015-0002-x. 25890379 10.1186/s13002-015-0002-x PMC4428097

[CR15] Emboden WA. Cannabis - a polytypic genus. Econ Bot. 1974;28:304–10. 10.1007/BF02861427.

[CR17] Fayvush G, Aleksanyan A, Bussmann RW. Ethnobotany of the Caucasus – Armenia. In: Bussmann R, editor. Ethnobot. Caucasus. Eur. Ethnobot. Cham: Springer; 2017. p. 21–36. 10.1007/978-3-319-49412-8_18.

[CR18] Grossheim AA. Plant wealth of the Caucasus. Moskovskoe obshchestvo ispytatelei prirody Publ., Moscow; 1952. (In Russian).

[CR19] Hanazaki N, Pieroni A, Ludwinsky RH, Gonçalves MC, Prakofjewa J, Peroni N, et al. People’s migrations and plants for food: a review for fostering sustainability. Sustain Earth Rev. 2023;6:9. 10.1186/s42055-023-00058-3.

[CR20] Hillig KW. Genetic evidence for speciation in *Cannabis* (Cannabaceae). Genet Resour Crop Evol. 2005;52:161–80. 10.1007/s10722-003-4452-y.

[CR21] Hourfane S, Mechqoq H, Bekkali AY, Rocha JM, El Aouad N. A Comprehensive Review on *Cannabis sativa* Ethnobotany, Phytochemistry, Molecular Docking and Biological activities. Plants. 2023;12:1245. 10.3390/plants12061245. 36986932 10.3390/plants12061245 PMC10058143

[CR22] Hovhannisyan LA. The history of medicine in Armenia. 2nd ed. Publishing House of AS of Arm. SSR, Yerevan; 1945. (In Russian).

[CR24] Hovsepyan R, Stepanyan-Gandilyan N. Use of plants in the folk medicine of the molokans of Armenia: preliminary data. Etnografia. 2021;2021:98–117. 10.31250/2618-8600-2021-2(12)-.

[CR25] Hovsepyan R, Stepanyan-Gandilyan N, Melkumyan H. Plant cultivation and gathering practices in yezidis’ and kurds’ cultures (preliminary data). Hist J. 2014;3:148–62. (In Armenian).

[CR26] Hovsepyan R, Stepanyan-Gandilyan N, Melkumyan H, Harutyunyan L. Food as a marker for economy and part of identity: traditional vegetal food of yezidis and kurds in Armenia. J Ethn Foods. 2016;3:32–41. 10.1016/j.jef.2016.01.003.

[CR27] Hovsepyan R, Stepanyan-Gandilyan N, Stollberg C. Phytomedicinal Knowledge and Official sources in Tatev (Armenia). Ethnobiol Lett. 2019;10:23–34. 10.14237/ebl.10.1.2019.1266.

[CR28] Hussain A, Abidi SH, Syed Q, Saeed A, Alim-Un-Nisa. Current knowledge on ethnobotany, phytochemistry and biological activities of *Cannabis* (hemp) from Pakistan with emphasis on its legalization and regulation. Ethnobot Res Appl. 2022;23:40.

[CR29] International Society of Ethnobiology. International Society of Ethnobiology Code of Ethics (with 2008 additions). 2008. www.ethnobiology.net/ethics.php

[CR30] Kharatyan-Arakelyan H. Armenian folk festivals. Publ. Zangak – 97, Yerevan; 2005. (In Armenian).

[CR31] Lamarck JB, Poiret JLM. Encyclopédie méthodique. Botanique., Paris, Panckoucke, Liège. Plomteux; 1783. 10.5962/bhl.title.824

[CR32] Lapierre É, Monthony AS, Torkamaneh D. Genomics-based taxonomy to clarify *Cannabis* classification. Genome. 2023;66:202–11. 10.1139/gen-2023-0005. 37163765 10.1139/gen-2023-0005

[CR33] Malabadi RB, Kolkar KP, Chalannavar RK. *Cannabis sativa*: Ethnobotany and Phytochemistry. Int J Innov Sci Res Rev. 2023;5:3990–8.

[CR34] McPartland JM. *Cannabis* Systematics alevelsLevels of Family, Genusspeciespecies. Cannabis Cannabinoid Res. 2018;3:203–12. 10.1089/can.2018.0039. 30426073 10.1089/can.2018.0039 PMC6225593

[CR35] Melkumyan IS. Wild edible plants of the Ararat kettle. Flora Veg Plant Resour Armen. 1991;13:228–47. (In Russian).

[CR36] Merriam-Webster D, Merriam-Webster. Dolma. 2024. https://www.merriam-webster.com/dictionary/dolma. Accessed 28 Mar 2024.

[CR37] Mnatsakanyan A. Armenian ornaments. Publishing House of the Academy of Sciences of the arm. SSR, Yerevan; 1955. (In Armenian).

[CR38] Muedi HTH, Kujoana TC, Shai K, Mabelebele M, Sebola NA. The use of industrial hemp (*Cannabis sativa*) on farm animal’s productivity, health and reproductive performance: a review. Anim Prod Sci. 2024;64. 10.1071/AN23268.

[CR39] Nanagulyan S, Zakaryan N, Kartashyan N, Piwowarczyk R, Łuczaj Ł. Wild plants and fungi sold in the markets of Yerevan (Armenia). J Ethnobiol Ethnomed. 2020;16:26. 10.1186/s13002-020-00375-3. 32429968 10.1186/s13002-020-00375-3 PMC7236950

[CR40] Nimmala M, Ross SD, Foroutan H. *Cannabis* pollen dispersal across the United States. Sci Rep. 2024;14(1):20605. 10.1038/s41598-024-70633-x. 39232057 10.1038/s41598-024-70633-x PMC11375005

[CR42] Ona G, Balant M, Bouso JC, Gras A, Vallès J, Vitales D, et al. The Use of *Cannabis sativa* L. for Pest Control: from the Ethnobotanical Knowledge to a systematic review of experimental studies. Cannabis Cannabinoid Res. 2022;7:365–87. 10.1089/can.2021.0095. 34612729 10.1089/can.2021.0095 PMC9418361

[CR43] Oregon CBD. Feminized seed and the ethics of Cannabis farming. Oregon CBD. 2017. https://ucanr.edu/sites/SoCo/files/316187.pdf. Accessed 21 Dec 2024.

[CR44] Petrov VA. Ethnobotany of Nagorno-Karabakh. Baku: Publ. FAS AZ; 1940. (In Russian).

[CR45] Pieroni A, Hovsepyan R, Manduzai AK, Sõukand R. Wild food plants traditionally gathered in central Armenia: archaic ingredients or future sustainable foods? Environ Dev Sustain. 2021;23:2358–81. 10.1007/s10668-020-00678-1.

[CR46] Rapetti L, Colombini S, Battelli G, Castiglioni B, Turri F, Galassi G, et al. Effect of Linseeds and Hemp seeds on milk production, Energy and Nitrogen Balance, and methane emissions in the dairy Goat. Animals. 2021;11:2717. 10.3390/ani11092717. 34573683 10.3390/ani11092717 PMC8470940

[CR47] Ren G, Zhang X, Li Y, Ridout K, Serrano-Serrano ML, Yang Y, et al. Large-scale whole-genome resequencing unravels the domestication history of *Cannabis sativa*. Sci Adv. 2021;7:eabg2286. 10.1126/sciadv.abg2286. 34272249 10.1126/sciadv.abg2286 PMC8284894

[CR48] Rivera D, Séiquer GM, Obón C, Ariza FA. Plants and humans in the Near East and the Caucasus. Ancient and traditional uses of plants as food and medicine, a diachronic ethnobotanical review. Murcia: Universidad De Murcia, 2 vol.; 2011.

[CR49] Rupasinghe HPV, Davis A, Kumar SK, Murray B, Zheljazkov VD. Industrial Hemp (*Cannabis sativa* subsp. *sativa*) as an emerging source for Value-Added Functional Food Ingredients and nutraceuticals. Molecules. 2020;25:4078. 10.3390/molecules25184078. 32906622 10.3390/molecules25184078 PMC7571072

[CR50] Russo E. *Cannabis* in India: ancient lore and modern medicine. In: Cannabinoids as therapeutics. Basel: Birkhäuser Basel; 2005. p. 1–22.

[CR51] Sargsyan M. Usage of spicy aromatic plants of the flora of Armenia in the national cuisine. Regul Mech Biosyst. 2023;14:469–75. 10.15421/10.15421/022367.

[CR52] Schultes RE, Klein WM, Plowman T, Lockwood TE, Cannabis. An example of taxonomic neglect. Bot Mus Lealf Harv Univ. 1974;23:337–67. 10.1515/9783110812060.

[CR53] Shakil SSM, Gowan M, Hughes K, Azam MNK, Ahmed MN. A narrative review of the ethnomedicinal usage of *Cannabis sativa* Linnaeus as traditional phytomedicine by folk medicine practitioners of Bangladesh. J Cannabis Res. 2021;3:8. 10.1186/s42238-021-00063-3. 33741060 10.1186/s42238-021-00063-3 PMC7980557

[CR54] Small E, Antle T. A preliminary study of pollen dispersal in *Cannabis sativa* in relation to wind direction. J Ind Hemp. 2003;8(2):37–50. 10.1300/J237v08n02_03.

[CR55] SOCIES Expert Center. Social research ethical guidelines: A practical guide. Yerevan. 2021. https://epfarmenia.am/sites/default/files/Document/DATA_Socies_Research_Ethics_Manual.pdf. (In Armenian, Accessed 22 Dec 2024).

[CR56] Srvandztyants G. Manana. Collect. Ed. Vol. 1, Publishing House of the Academy of Sciences of the Arm. Yerevan: SSR; 1876. p. 117–364 (In Armenian).

[CR57] Stepanyan- Gandilyan NP. Ethnobotanical studies of the Armenian Highland: past and future. Yerevan, Gitutyun; 2014. (In Russain).

[CR59] Stoletova EA. Field and vegetable crops of Armenia. Bull Appl Bot Genet Plant-Breeding. 1930;24:1–376. (In Russian).

[CR60] Vardanyan AV. Interdialect of Vayots dzor. Tigran Mets Publ., Yerevan; 2004, 509 pp. (In Armenian) https://language.sci.am/sites/default/files/book/vardanyan_vayots_dzor.pdf.pdf . 28 Oct 2024, date last accessed.

[CR61] Vulf YV. *Cannabis*. main Crop plants Descr. Orig., Selkhozgiz, Moscow; 1940, p. 101–103. (In Russian).

[CR62] Xie Z, Mi Y, Kong L, Gao M, Chen S, Chen W, et al. *Cannabis sativa*: origin and history, glandular trichome development, and cannabinoid biosynthesis. Hortic Res. 2023;10:uhad150. 10.1093/hr/uhad150. 37691962 10.1093/hr/uhad150 PMC10485653

[CR63] Yatsenko-Khmelevskiy AA. Cannabaceae. In: Takhtajan AL, editor. Flower. plants. Plant life, Prosvescheniye, Moscow, 1; 1980, p. 279–281. (In Russian).

[CR64] Yeznik K. Refutation of the sects. Translated from Old Armenian (Grabar) and commented by A. A. Abrahamyan. YSU Publishing House, Yerevan; 1994. (In Armenian).

[CR65] Zhukovskiy PM. Cultivated plants and their wild relatives. 3rd ed. Leningrad, USSR, Kolos; 1971.

[CR66] Zhukovskiy PM. Fam. Cannabiaceae. Bot. 5th ed. Moscow: Kolos; 1982. pp. 430–1. (In Russian).

[CR67] Zou S, Kumar U. Cannabinoid receptors and the Endocannabinoid System: signaling and function in the Central Nervous System. Int J Mol Sci. 2018;19:833. 10.3390/ijms19030833. 29533978 10.3390/ijms19030833 PMC5877694

