# Supplementary material for: Bridging past and present: exploring Cannabis traditions in Armenia through ethnobotanical interviews and bibliographic prospecting
Source: J Cannabis Res. 2025 Jan 30;7:8. doi: 10.1186/s42238-025-00259-x (PMC11780856; doi:10.1186/s42238-025-00259-x)
Supplement: Supplementary file 1 — Supplementary Material 1. [file 42238_2025_259_MOESM1_ESM.pdf]

Ethnobotanical questionnaire about the use of hemp (*Cannabis sativa*)

Էթնոբուսաբանական հարցարան կանեփի (*Cannabis sativa*) օգտագործման վերաբերյալ

For each question the following information will be collected as completely as possible:
Յուրաքանչյուր հարցի շուրջ անհրաժեշտ է հավաքել հնարավորինս ամփոփ տեղեկություններ.

- Part of the plant used (given name in every case) (used language)
- Բույսի օգտագործվող մասը (յուրաքանչուր դեպքում նշել անունը) (օգտագործվող լեզուն)
- Finality
- Վերջնարդյունքը
- How it is prepared
- Պատրաստման եղանակը
- How it is administered, applied or used.
- Ինչպե՞ս է բաշխվում, գործածվում կամ կիրառվում:

All the information given about the plant and its uses will be collected, even if it may seem irrelevant (collection and conservation methods, how the information has been accessed, observations on physical aspects of the plant, opinions and personal observations on the plant and its uses and management, etc.).

Բույսի և դրա օգտագործման մասին ստացված ամբողջ ինֆորմացիան գրանցվում է, նույնիսկ այն դեպքում, եթե այն կարող է թվալ թեմային չվերաբերող (բույսը հավաքելու, պահելու մեթոդները, որտեղի՞ց են ծանոթ այդ մեթոդներին, բույսի արտաքին տեսքի մասին դիտարկումները, բույսի մասին կարծիքները և անձնական դիտողությունները դրա օգտագործման, կիրառման ոլորտների մասին և այլն):

Data about the interview
Տեղեկություններ հարցազրուցի մասին

Locality:<br>Որտե՞ղ.	Date:<br>Ե՞րբ.
Language of the interview:<br>Ի՞նչ լեզվով է անցկացվում հարցազրույցը.	
Language of the informant:<br>Ինֆորմանտի (տեղեկություններ հաղորդողի) լեզուն	
Was an interpreter necessary? YES / NO<br>(If yes, from which language to which language)<br>Թարգմանչի անհրաժեշտությունը. ԱՅՈ / ՈՉ<br>(Եթե այո, ապա ո՞ր լեզվից ո՞ր լեզվին)	

Data about the informant
Տեղեկություններ ինֆորմանտի մասին

Name of the interviewee:<br>Անունը.
Sex:<br>Սեռը.
Year of birth:<br>Ծննդյան թիվը.
Place of birth:<br>Ծննդավայրը.
Places of residence along life (and years of permanence in each):<br>Ապրելավայրերը կյանքի ընթացքում՝ տարիներով<br>(և տևողությունը յուրաքանչյուր տեղում).

Questionnaire

Հարցաշար

1. Common names of the plant, of its used parts and of its derived products (annotate the used language):

1. Բույսի ժողովրդական անունը, դրա օգտագործվող մասերի և ստացվող ապրանքի անվանումները (նշել օգտագործվող լեզուն):

**Name**<br>Անվանումը	**Language**<br>Լեզուն

2. Medicinal finalities of the plant (used part, finality, preparation, way of administration, destination to human and/or veterinary medicine)

2. Բուժիչ նպատակով բույսի կիրառումը (օգտագործվող մասերը, վերջնարդյունքը, կիրառման եղանակը, նշանակությունը մարդու համար կամ անասնաբուժության մեջ)

**Used part**<br>Օգտագործվող մասը	**Finality**<br>Վերջնարդյունքը	**Preparation**<br>Պատրաստման եղանակը	**Way of administration**<br>Կիրառման եղանակը	**Destination**<br>Նպատակը

3. Food finalities of the plant (used part, finality, preparation, destination to human food and/or animal feed)

3. Ուտելու նպատակով բույսի կիրառման վերջնարդյունքը (օգտագործվող մասերը, նպատակը, կիրառման եղանակը, նշանակությունը որպես մարդու սննունդ կամ անասնակեր)

**Used part**<br>Օգտագործվող մասը	**Finality**<br>Վերջնարդյունքը	**Preparation**<br>Պատրաստման եղանակը	**Destination**<br>Նպատակը

4. Other uses of the plant: textile, for paper production, in building, in elaboration of tools, instruments, etc. (used part, finality, preparation)

4. Այլ նպատակներով բույսի կիրառումը՝ գործվածք կան թուղթ ստանալու համար, շինարարություն, գործիքների պատրաստում (օգտագործվող մասերը, նպատակը, պատրաստման եղանակը)

**Used part**<br>Օգտագործվող մասը	**Finality**<br>Վերջնարդյունքը	**Preparation**<br>Պատրաստման եղանակը

5. Is hemp smoked in your territory? YES / NO

5. Օգտագործվո՞ւմ է արդյոք կանեփը ծխելու համար Ձեր տարածաշրջանում: ԱՅՈ / ՈՉ

6. If yes, with what objective? (used part, finality, preparation)

6. Եթե այո, ապա ինչպե՞ս է օգտագործվում (օգտագործվող մասերը, վերջնարդյունքը, պատրաստման եղանակը)

**Used part**<br>Օգտագործվող մասը	**Finality**<br>Վերջնարդյունքը	**Preparation**<br>Պատրաստման եղանակը

7. Is hemp considered a sacred or venerated plant in your territory? YES / NO

7. Դիտարկվո՞ւմ է արդյոք կանեփը որպես սուրբ կամ պաշտվող բույս Ձեր տարածաշրջանում. ԱՅՈ / ՈՉ

8. Is there any ritual associated to this plant? YES / NO

8. Կա՞ արդյոք որևէ ծես/ արարողություն՝ կանեփի հետ կապված: ԱՅՈ / ՈՉ

9. If yes, describe it (used part, finality, preparation)

9. Եթե այո, նկարագրեք այն (օգտագործվող մասերը, վերջնարդյունքը, պատրաստման եղանակը)

**Used part** Օգտագործվող մասերը	**Finality** Վերջնարդյունքը	**Preparation** Պատրաստման եղանակը

10. Is there any saying or any song associated to this plant? YES / NO

10. Կա՞ արդյոք որևէ ժողովրդական ասացվածք, երգ՝ կանեփի հետ կապված: ԱՅՈ / ՈՉ

11. If yes, mention and describe it/them

11. Եթե այո, ներկայացրեք կամ նկարագրեք այն:

12. Is this plant used in any game? YES / NO

12. Որևէ խաղում այդ բույսը օգտագործվո՞ւմ է: ԱՅՈ / ՈՉ

13. If yes, describe it (used part, finality)

13. Եթե այո՝ նկարագրեք այն (օգտագործվող մասերը, նպատակը)

**Used part** Օգտագործվող մասերը	**Finality** Նպատակը

14. Where have the seeds used for the present plantation been obtained?

14. Որտեղի՞ց են ստացվել սերմերը տվյալ ցանքի համար:

15. Since when are you cultivating the same seed?

15. Երբվանի՞ց եք օգտագործում այդ սերմերը:

16. How do you distinguish between male and female plants?

16. Ինչպե՞ս եք տարբերում արական և իգական բույսերը:

17. Is all the hemp you know cultivated? YES / NO

17. Միայն մշակո՞վի կանեփն է Ձեզ ծանոթ: ԱՅՈ / ՈՉ

18. Do you perform any kind of agricultural practice associated to hemp's cultivation? YES / NO

18. Կանեփը մշակելու համար որևէ հատուկ գյուղատնտեսական մեթոդներ օգտագործո՞ւմ եք: ԱՅՈ / ՈՉ

19. If yes, describe it

19. Եթե այո՝ նկարագրեք այն:

20. When are the seeds sown?

20. Ե՞րբ էին ցանած այդ սերմերը:

21. When are the plants harvested?

21. Ե՞րբ են հավաքում բույսերը:

22. Did you use wild hemp? YES / NO

22. Վայրաճ կանեփը օգտագործո՞ւմ եք: ԱՅՈ / ՈՉ

23. If yes, where do you collect it?

23. Եթե այո՝ որտե՞ղ եք այն հավաքում:

24. Could you show or indicate where the plant grows wild? YES / NO

24. Կարող եք ցույց տալ կամ նշել այդ բույսի վայրի աճման տեղերը: ԱՅՈ / ՈՉ

25. Do you distinguish more than a type of hemp? YES / NO

25. Նկատե՞լ եք կանեփի տարբեր ձևեր: ԱՅՈ / ՈՉ

26. If yes, do you perform any crossing between the different types of hemp? YES / NO

26. Եթե այո՝ փորձե՞լ եք այդ տարբեր ձևերը տրամախաչել: ԱՅՈ / ՈՉ

27. If yes, describe it

27. Եթե այո՝ նկարագրեք:

28. Since when do you know the plant?

28. Երբվանի՞ց է Ձեզ ծանոթ այդ բույսը:

29. Who explained you its uses?

29. Ո՞վ է Ձեզ պատմել դրա օգտագործման մասին:

30. Other information (how is it preserved, etc.)

30. Այլ տեղեկություններ (պահելու եղանակները և այլն):
